# Defibrotide modulates pulmonary endothelial cell activation and protects against lung inflammation in pre-clinical models of LPS-induced lung injury and idiopathic pneumonia syndrome

**DOI:** 10.3389/fimmu.2023.1186422

**Published:** 2023-06-27

**Authors:** Orly R. Klein, Yiouli P. Ktena, Elizabeth Pierce, Han-Hsuan Fu, Azeb Haile, Chen Liu, Kenneth R. Cooke

**Affiliations:** ^1^ Department of Oncology, Pediatric Blood and Marrow Transplant Program, The Sidney Kimmel Comprehensive Cancer Center at Johns Hopkins, Baltimore, MD, United States; ^2^ Department of Pediatrics, Pediatric Blood and Marrow Transplant Program, Case Western Reserve University, School of Medicine, Cleveland, OH, United States; ^3^ Department of Pathology, Yale School of Medicine, New Haven, CT, United States

**Keywords:** pulmonary, inflammation, vascular endothelium, allogeneic bone marrow transplantation, murine models, cytokines

## Abstract

**Introduction:**

A multiple organ dysfunction syndrome (MODS) workshop convened by the National Institute of Child Health and Human Development in 2015 identified acute respiratory distress syndrome (ARDS) and complications of allogeneic blood and marrow transplantation (allo-BMT) as contributors to MODS in pediatric patients. Pulmonary dysfunction also remains a significant complication of allo-BMT. Idiopathic pneumonia syndrome (IPS) defines non-infectious, acute, lung injury that occurs post-transplant. Injury and activation to endothelial cells (ECs) contribute to each form of lung inflammation.

**Methods:**

Two murine models were employed. In an ARDS model, naïve B6 mice receive an intravenous (i.v.) injection of lipopolysaccharide (LPS). In the established model of IPS, naïve B6D2F1 mice receive lethal total body irradiation followed by BMT from either allogeneic (B6) or syngeneic (B6D2F1) donors. Lung inflammation was subsequently assessed in each scenario.

**Results:**

Intravenous injection of LPS to B6 mice resulted in enhanced mRNA expression of TNFα, IL-6, Ang-2, E-, and P-selectin in whole lung homogenates. The expression of Ang-2 in this context is regulated in part by TNFα. Additionally, EC activation was associated with increased total protein and cellularity in broncho-alveolar lavage fluid (BALF). Similar findings were noted during the development of experimental IPS. We hypothesized that interventions maintaining EC integrity would reduce the severity of ARDS and IPS. Defibrotide (DF) is FDA approved for the treatment of BMT patients with sinusoidal obstruction syndrome and renal or pulmonary dysfunction. DF stabilizes activated ECs and protect them from further injury. Intravenous administration of DF before and after LPS injection significantly reduced mRNA expression of TNFα, IL6, Ang-2, E-, and P-selectin compared to controls. BALF showed decreased cellularity, reflecting less EC damage and leak. Allogeneic BMT mice were treated from day -1 through day 14 with DF intraperitoneally, and lungs were harvested at 3 weeks. Compared to controls, DF treatment reduced mRNA expression of TNFα, IL6, Ang-2, E-, and P- selectin, BALF cellularity, and lung histopathology.

**Conclusion:**

The administration of DF modulates EC injury in models of ARDS and IPS. Cytokine inhibition in combination with agents that stabilize EC integrity may be an attractive strategy for patients in each setting.

## Introduction

Allogeneic blood and marrow transplantation (allo-BMT) is the only curative option for many pediatric, adolescent, and young adult patients with malignant and nonmalignant disorders (1, 2). Unfortunately, treatment-related complications continue to limit successful outcomes and opportunities to broaden the scope of therapy. A multiple organ dysfunction syndrome (MODS) workshop convened by the Eunice Kennedy Shriver National Institute of Child Health and Human Development in March of 2015 identified acute respiratory distress syndrome (ARDS) and complications associated with allo-BMT as distinct contributors to MODS and death in pediatric patients (3, 4). The significance of respiratory failure occurring after BMT was further underscored by a June 2018 National Institute of Health (NIH) workshop specifically convened to identify clinical challenges and scientific knowledge gaps facing pediatric BMT patients with lung injury (5). Hence, the development of novel strategies to reduce the incidence and severity of pulmonary dysfunction, or treat it once developed, remains one of the greatest challenges to the optimization of care following allo-BMT.

Almost half of the pneumonias after allo-BMT are noninfectious in origin, and are defined as idiopathic pneumonia syndrome (IPS) when occurring early after transplant and in the absence of heart failure and iatrogenic fluid overload (5, 6). Clinical and experimental IPS are associated with evidence for pulmonary vascular injury and leak, as demonstrated by the presence of pulmonary edema, enhanced total protein levels in broncho-alveolar lavage fluid (BALF), and increased wet-to-dry lung weight ratios (6, 7). Using an established murine model, we previously demonstrated that significant endothelial cell (EC) apoptosis correlates with the severity of lung histopathology 6 weeks after allo-BMT (8). Moreover, apoptotic pulmonary ECs are present at early stages of leukocyte infiltration and are associated with EC activation, as evidenced by enhanced mRNA expression of adhesion molecules (VCAM, ICAM, and E-selectin) and elevated levels of TNFα in BALF (8). We and others have demonstrated that soluble proteins, including cytokines and chemokines, which have been shown to mediate inflammation responsible for clinical and experimental ARDS (9), also contribute to the development of IPS in mice and humans (6, 10–13).

Importantly, EC damage and dysfunction likely represents a common-thread among ARDS and several allo-BMT-related complications, including IPS, graft versus host disease (GVHD), thrombotic microangiopathy, and veno-occlusive disease (VOD)/sinusoidal obstruction syndrome (SOS) of the liver (14–17), all of which can contribute to the MODS frequently seen after transplantation (3, 4). Independent biomarker data also suggest that biologic pathways contributing to EC injury and leak during IPS (6, 18–20) are likely operative during the development of GVHD and SOS (6, 21–25), along with lung injury that associates with infectious and non-infectious ARDS outside of the BMT setting.

**Figure 1:**
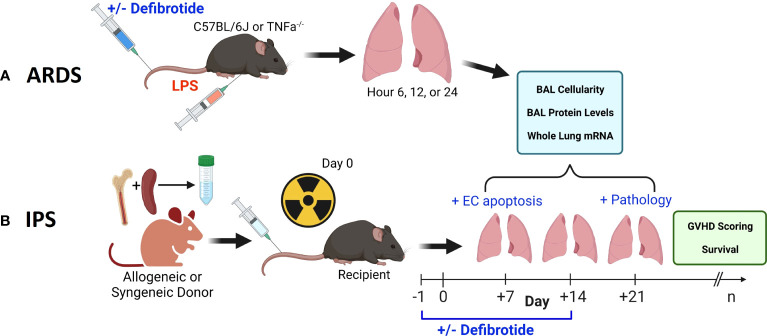
Two pre-clinical, murine, models were employed in this work. An ARDS model wherein naïve B6 mice received an intravenous (i.v.) injection of LPS **(A)** and an established, model of IPS wherein naïve, B6D2F1 mice received lethal total body irradiation followed by BMT from either allogeneic (B6) or syngeneic (B6D2F1) donors **(B)**. We first demonstrate that the injurious stimulus (LPS vs. allogeneic BMT) results in reproducible lung inflammation as measured by mRNA expression along with BAL cellularity and protein content. In each scenario, the protective effects of defibrotide (DF) were assessed in subsequent experiments. In the BMT model, the impact of DF administration on endothelial cell apoptosis (Day 7), lung pathology (D21) along with survival and clinic GVHD score was determined.

Hence, strategies that protect EC integrity may lessen lung injury and dysfunction in several scenarios, and therefore have significant scientific and clinical merit. Defibrotide (DF) is an agent FDA approved for the treatment of patients who develop VOD/SOS after BMT that is associated with either renal or pulmonary dysfunction. Importantly, DF has been shown to have properties that stabilize and protect ECs from injury and activation (26–29). We therefore explored the effects of DF administration in established models of BMT and non-BMT acute lung injury to determine whether DF can modulate markers of lung inflammation and EC activation (**Figure 1**). Our results demonstrate that the administration of DF reduces pulmonary inflammation and capillary leak in each setting, and suggest that agents which stabilize EC integrity may be attractive therapeutic options for patients with ARDS and IPS.

## Methods

### Mice and bone marrow transplant

Female mice (B6D2F1/J (F1; H-2^bxd^) and C57BL/6J (B6; H-2^b^), along with C57B6-*Tnfrsf1a^tm1Imx^*/J (p55ko), B6.129S- *Tnfrsf1b^tm1Imx^* (p75ko) and B6.129S-*Tnfrsf1b^tm1Imx^ Tnfrsf1a^tm1Imx^*/J (p55/p75 DKO) were purchased from Jackson Laboratories (Bar Harbor, ME). C57BL/B6 TNFα^-/-^; C57BL/B6 IL-6^-/-^ were backcrossed for 12 generations and bred in the Cooke laboratory mouse colony at Johns Hopkins University School of Medicine. Animals used for all *in vivo* experiments were between 8 and 14 weeks of age. Mice received BMT as previously described (13, 30, 31). In brief, the spleens, tibias, and femurs were harvested from donor mice. Single cell suspensions of BM (femurs and tibias) and spleen cells were made by gentle mechanical dissociation. T cell purification from splenic cells was performed by magnetic bead separation using an AutoMACS Pro (Miltenyi, Auburn, CA) instrument and CD90.2 positive selection. Purity of splenic T cell fraction was consistently in the 80% to 95% range as confirmed by flow cytometry using antibodies to CD3. Cell mixtures of 5 x 10^6^ BM cells and 2 x 10^6^ splenic T cells were suspended in 0.25 ml of Leibovitz media prior to injection. Recipient B6D2F1 mice received 13 Gy total body irradiation (TBI) in two fractions separated by four hours to reduce intestinal toxicity. Radiation was delivered by X-strahl CIXD (Suwanee, GA) dual-headed photon irradiator. The donor BM and T cell inoculum from either allogeneic C57BL/6 (B6) or syngeneic B6D2F1 (F1) donors was administered via tail vein injection. Mice were housed in pathogen-free cages, administered acidified water, and monitored frequently as described below. BMT parameters for this model result in approximately 60% mortality by D49 (32, 33) and the development of significant, reproducible, lung injury and GVHD in surviving animals (13, 30, 31). All experiments were approved by the JHU Animal Care and Use Committee protocol numbers MO13M346, MO16M262, and MO19M300.

### Terminal exsanguination

At the time of final evaluation, mice were anesthetized with isoflurane in an enclosed chamber. Anesthesia was confirmed by assessing lack of response to pain. Mice were secured for terminal exsanguination from the retro-orbital plexus, and blood collected into a 1.5 mL Eppendorf tube. Blood was centrifuged at 10000 revolutions per minute (rpm) for 10 minutes, and serum was collected and frozen for future analysis.

### Grading of systemic and pulmonary GVHD severity

In BMT experiments, mice were ear tagged and weighed prior to BMT, and then monitored longitudinally for survival (daily) and clinical signs of GVHD (weekly), using an established clinical scoring system that includes 5 parameters: weight loss, fur texture, skin integrity, posture, and activity (34). At specified time points, lung tissue was collected, formalin fixed, and paraffin embedded before tissue sections were prepared and stained with hematoxylin and eosin. Tissues from individual mice were coded without reference to mouse type or treatment regimen, and independently examined and graded according to severity, pattern, and extent of injury, as previously described (30).

### Broncho-alveolar lavage and lung harvest

After terminal exsanguination, BAL was performed as previously described (13, 31, 34). In brief, the neck was dissected and connective tissue around the trachea was removed. A small incision was made in the trachea, and a blunt-end needle was inserted into the trachea, and secured in place with thread. 0.8mls of Phosphate buffered saline (PBS) containing ethylenediaminetetraacetic acid (EDTA) was instilled into the airways via the trachea and then removed, and collected into tubes. This process was repeated a total of 4 times. The BAL fluid (BALF) was spun at 1400 rpm at 4 degrees Celsius. After centrifugation, supernatants from the first lavage were frozen for subsequent analysis of protein and cytokine content, and cell pellets from all lavages were washed in PBS three times, and counted. After BAL was completed, the thoracic cage was opened to expose the heart and lungs. PBS was injected into the right atrium to flush the pulmonary vasculature. The lungs were dissected and stored for additional testing. One or several of the lobes were either (a) fixed in formalin, and paraffin embedded for subsequent slide preparation and histopathologic review; (b) snap frozen, and then homogenized and RNA extracted for RT-PCR; (c) placed in PBS, then homogenized for protein extraction and subsequent ELISA or (d) placed in PBS, then homogenized for endothelial cell separation (see below).

### Reverse transcription polymerase chain reaction

Frozen lung tissue was homogenized following a manufacture’s protocol for “homogenization of tissue for total RNA isolation” (Miltenyi, Auburn, CA). Lungs were placed in an M Tube along with buffer RLT (Qiagen, Hilden, Germany) and homogenized using a gentleMACS tissue dissociator (Miltenyi). RNA was extracted using RNEasy kit and protocol (Qiagen), including an on-column DNAse digestion. RNA was converted to cDNA using iSCript cDNA synthesis kit (Bio-Rad, Hercules, CA). RT-PCR was performed using TaqMan Universal PCR MasterMix and the designated Taqman primers (Thermo Fisher) and run on a CFX96 Touch Real-Time PCR Detection System (Bio-Rad).

### Protein extraction

Lung lobes were harvested, as described above, and placed in PBS on ice. Lungs were placed in M tubes with Pierce RIPA buffer, Halt Protease Inhibitor cocktail, and Halt Protease & Phosphatase Inhibitor Cocktail (Thermo Scientific), and tissue was homogenized using a gentleMACS tissue dissociator (Miltenyi). Supernatant was either analyzed immediately or frozen for future use.

### Enzyme-linked immunosorbent assay

Protein concentrations were measured in batch on stored serum, BALF, and protein extracted from homogenized lung tissue samples. Concentration of total protein was measured using Pierce BCA protein assay (Thermo Scientific, Waltham, MA). Concentrations of specific cytokines were measured using ELISA kits for Angiopoietin-2 and TPA (Abcam, Cambridge, United Kingdom). Assays were performed according to the manufacturer’s protocol. Assay sensitivity was less than 5 ug/mL for whole protein, 4.98 pg/mL for Ang-2 and 4.98 pg/ml for TPA. ELISA plates were read on an Emax Microplate reader (Molecular Devices, San Jose, CA).

### Lipopolysaccharide-induced ARDS

Female C57BL/6J mice were injected intravenously with lipopolysaccharide (LPS), *Escherichia coli* serotype 026:B6 (Sigma, St. Louis, MO). LPS 500 ug was dissolved in 0.25 mL PBS. Mice were sacrificed via terminal exsanguination at specified time points, and further analyses were performed. In some experiments mice deficient in TNFα (H-2^b^; TNFα-/-) or in the TNFα p55 receptor C57B6-*Tnfrsf1a^tm1Imx^*/J (p55ko), the p75 receptor B6.129S*-Tnfrsf1b^tm1Imx^* (p75ko) (35) or both B6.129S-*Tnfrsf1b^tm1Imx^ Tnfrsf1a^tm1Imx^*/J (p55/p75 DKO) were used in LPS-induced, acute lung injury experiments.

### Defibrotide

Defibrotide, supplied in powder under a material transfer agreement with Jazz pharmaceuticals (Palo Alto, CA), was measured by dry weight on a Mettler Toledo MS54S Balance (Columbus, OH). The DF powder was dissolved in sterile PBS using a vortex mixer to yield a concentration of 12.5 mg/ml. This was all performed in a sterile hood. Defibrotide solution was either used immediately, or stored at 4 degrees C for up to one week prior to administration. Dose finding studies were performed as follows: mice received 25 mg/kg or 50 mg/kg I.V., and 25 mg/kg, 50 mg/kg, or 100 mg/kg intraperitoneally (i.p.). Mice were terminally exsanguinated at 30 minutes, 60 minutes, and 120 minutes after DF dose. Blood samples were collected into BD microtainer (Franklin Lakes, NJ) Lavender EDTA tubes, and centrifuged at 10,000 rpms for 15 minutes. Plasma was transferred into a clean plastic tube and frozen. Samples were analyzed using a mouse TPA activity ELISA kit (Oxford Biomedical Research, Rochester Hills, MI). In LPS challenge experiments, mice received DF or PBS i.v. one hour before and 5 hours after LPS exposure. In BMT experiments, mice received either DF or PBS control i.p. starting the day prior to BMT, through 14 days after BMT. DF was given either daily or twice daily in a divided dose.

### Endothelial apoptosis assay

We established an assay to examine evidence for EC apoptosis after BMT using flow cytometric measurements. Irradiated B6D2F1 mice underwent syn- or allo-BMT as described above. Animals were sacrificed at week 1 after BMT and lungs were procured and digested to create a single-cell suspension using a GentleMACS (Miltenyi Biotec) tissue dissociator and a digestion mixture containing collagenase IV (0.05%) and DNase (1mg/ml). In brief, the trachea was cannulated and secured with sutures as described above. Lungs were flushed with 10 mL of ice-cold DMEM. Next, 1 mL of dispase (Corning) was injected through the trachea and followed by 500 μL of liquid low gelling agarose (Sigma). Once agarose solidified, lungs were removed and incubated in 0.05% of collagenase IV (Gibco) plus 1 mg/mL of DNase (Sigma) for 10 mins at 37°C. After digestion, cell suspension was filtered through 70 μM strainer to achieve a single cell suspension. Cells were counted and subsequently stained for surface markers CD31 and CD45 (Biolegend) along with LIVE/DEAD Fixable Viability Dye (Invitrogen) for 30 mins at 4°C. Cells were next washed with PBS and then washed with Annexin V binding buffer following the manufacture protocol (BD Biosciences). Cells were subsequently stained with Annexin V (BioLegend) for 15 mins at room temperature. Finally, cells were resuspended in Annexin V binding buffer and prepared for FACS analysis. Alternatively, following incubation with antibodies to CD31, CD45 and LIVE/DEAD Fixable Viability Dye for 30 mins at 4°C, cells were washed with DMEM and then stained with CellEvent Caspase 3/7 according to manufacture protocol (Invitrogen) for 25 mins at 37°C. After a final wash with DMEM, cells were resuspended in PBS and prepared for FACS analysis.

Pulmonary ECs (CD31+) were distinguished from immune cells (CD45+) as being CD31+/CD45-. The presence of EC apoptosis was determined by staining for either Annexin V or caspase 3/7 together with cell viability dyes. Preliminary experiments demonstrated that lung EC cells (CD31+/CD45-) were extremely sensitive to mechanical dissociation and magnetic bead purification methods when compared to CD45+ immune cells; background levels of apoptosis were very high in lung EC of both syngeneic BMT recipients and naïve, un-transplanted mice (data not shown). Enzyme concentrations in the digestion mixture and incubation times were optimized in several pilot experiments to consistently show differences between the extent of pulmonary EC apoptosis observed in allo-BMT recipients compared to both syngeneic and naïve controls using annexin V and caspase 3/7.

### Statistical analysis

All values are expressed as the mean and the standard error of the mean (SEM). Statistical comparisons between groups were calculated using the parametric independent sample t test for experiments with 5 or more animals per group, or using the Mann-Whitney test for fewer than 5 animals per group. The Wilcoxon signed-rank test was used to analyze survival data.

## Results

### Systemic administration of LPS results in acute lung inflammation and capillary leak

**Figure 2:**
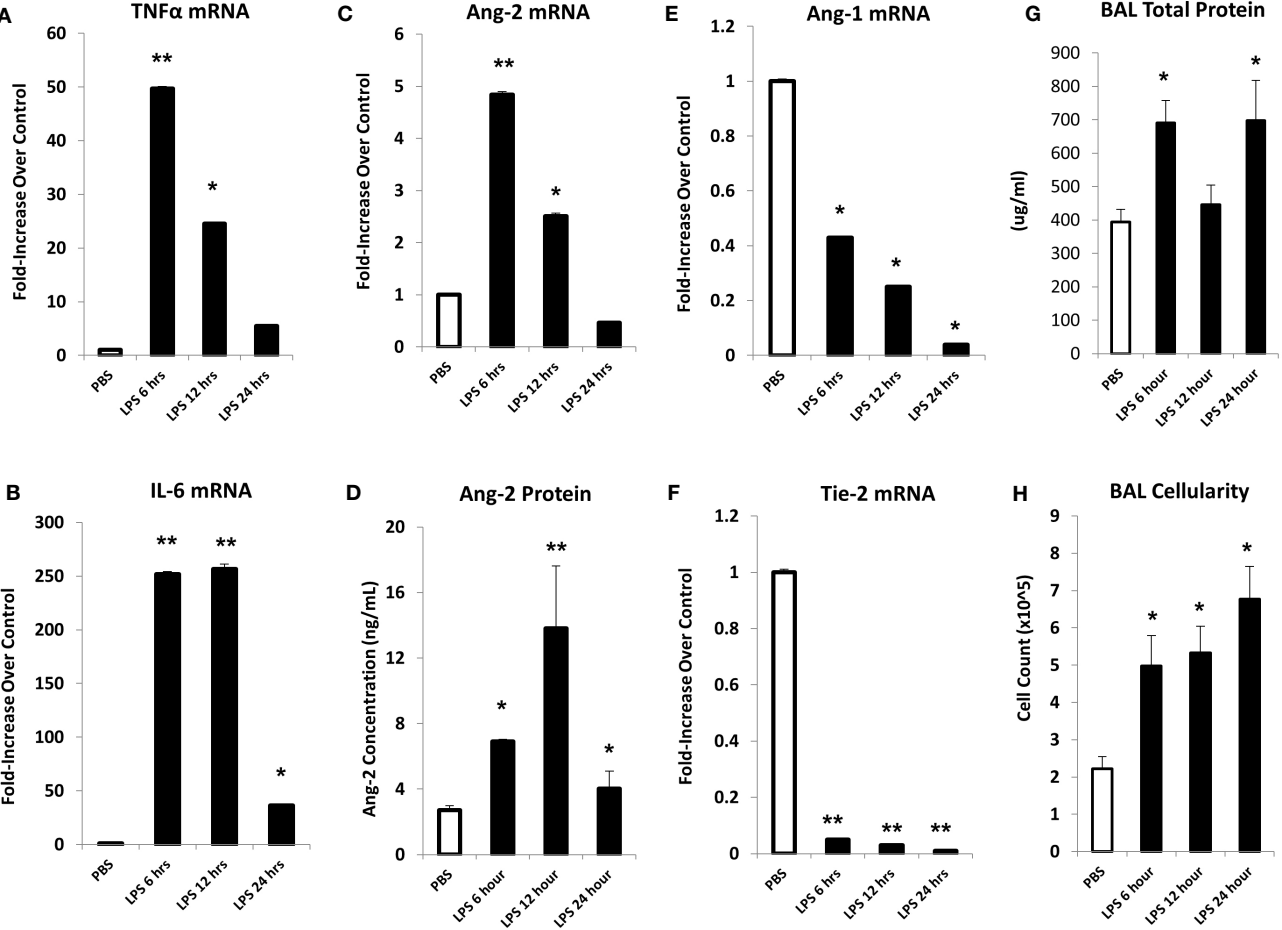
In an LPS-induced ARDS model, C57BL/6J mice were injected with LPS as described in *Materials and Methods* and subsequently analyzed at 6, 12, or 24 hours thereafter. Mice receiving PBS were analyzed at the 6-hour time point. mRNA analysis of whole lung tissue shows marked elevation in the expression of TNFα **(A)**, IL-6 **(B)**, and Ang-2 **(C)** with elevations of Ang-2 protein **(D)** as measured by ELISA. Marked reductions in Ang-1 **(E)** and Tie-2 **(F)** mRNA expression were also noted. Prior to lung extraction, mice underwent BAL as described in *Materials and Methods.* LPS injection results in elevations of BALF total protein levels **(G)** and cellularity **(H)** indicative of EC injury and leak (n = 4 mice per group; data are from one of three replicate experiments; *p < 0.05; **p < 0.01 compared to PBS group).

**Figure 3:**
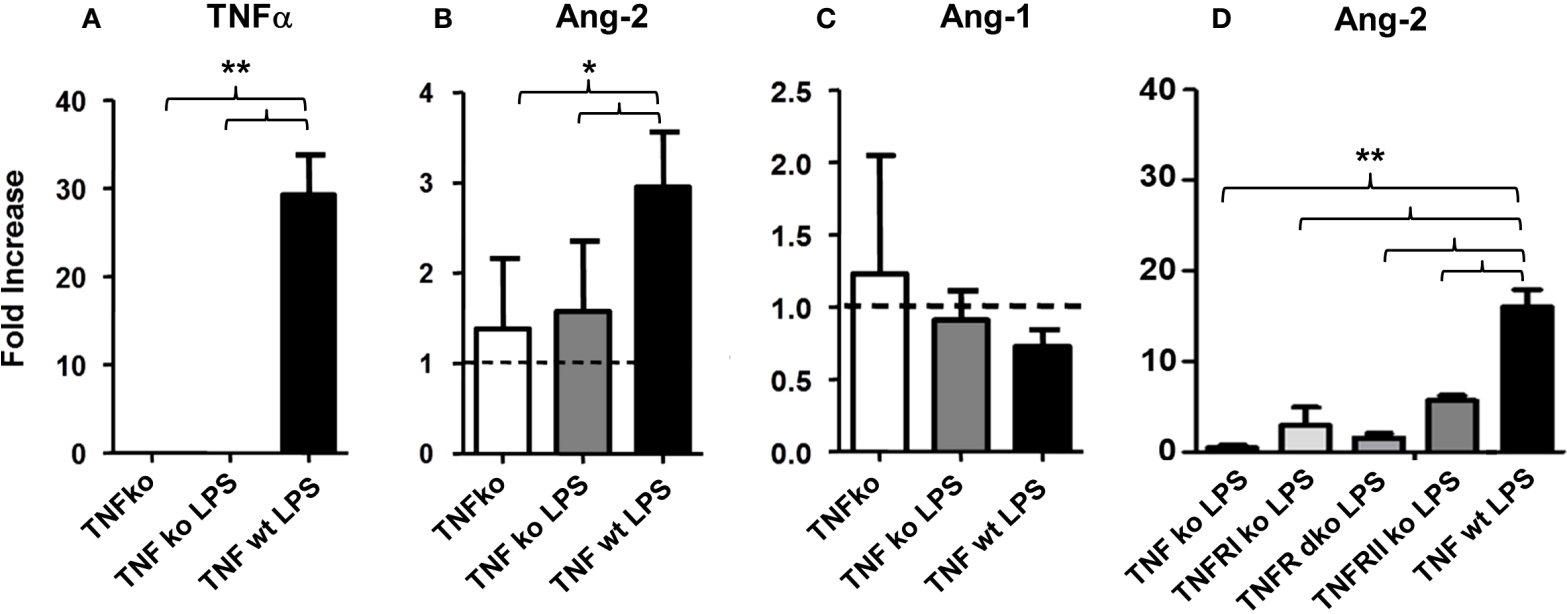
C57BL/6J mice, or mice deficient in TNFα, the TNFα p55 receptor (TNFRI), the p75 receptor (TNFRII), or both (TNFR dko) were injected with LPS as described in **Figure 2** and subsequently analyzed 6 hours later. Control animals received PBS. The administration of LPS to naïve B6 mice results in enhanced mRNA expression of TNFα **(A)** and Ang-2 **(B)** whereas Ang-1 expression is down regulated **(C)**. Ang-2 expression is regulated by TNFα production and presence of both TNF receptors **(B–D)** (n = 5 mice per group; data are from one of three replicate experiments; *p < 0.05, **p < 0.01).

In initial experiments, mice were injected with LPS or PBS, and lungs were harvested at 6, 12, or 24 hours after LPS exposure. Analysis of whole lung tissue revealed that mRNA expression of TNFα, IL-6, and Ang-2 was markedly elevated after LPS injection; expression was highest at the 6-hour time point, trending back to control levels by 24 hours (**Figures 2A–C**). Accordingly, whole lung Ang-2 protein was also elevated, peaking at the 12-hour time point, and returning to control levels by 24 hours (**Figure 2D**). By contrast, Ang-1 and Tie2 mRNA expression was lower in LPS exposed animals compared to controls (**Figures 2E, F**). BAL fluid total protein was elevated compared to controls 6- and 24-hours following injection (**Figure 2G**), and BAL cellularity was increased at each time point (**Figure 2H**) consistent with lung inflammation along with EC activation and leak observed in this model of ARDS. We next determined if inflammation engendered following LPS administration and associated effects on Ang-2 expression are significantly regulated by TNFα;TNF receptor interactions. C57BL/6J mice, or mice deficient in TNFα, the TNFα p55 receptor, the p75 receptor, or both were injected with LPS as described in **Figure 2** and subsequently analyzed 6 hours later. Control animals received PBS. As before, the administration of LPS to naïve B6 mice results in enhanced mRNA expression of TNFα (**Figure 3A**) and Ang-2 (**Figure 3B**) whereas Ang-1 expression is down regulated (**Figure 3C**). By contrast, Ang2 expression was significantly lower in mice deficient in TNFα or either TNFα receptor after injection with LPS (**Figures 3B, D**). Collectively, these observations revealed that Ang-2 expression is regulated by TNFα production and the presence of both TNF receptors.

### Experimental IPS is associated with increased inflammatory cytokine expression and evidence of EC activation and injury early after allo-BMT

**Figure 4:**
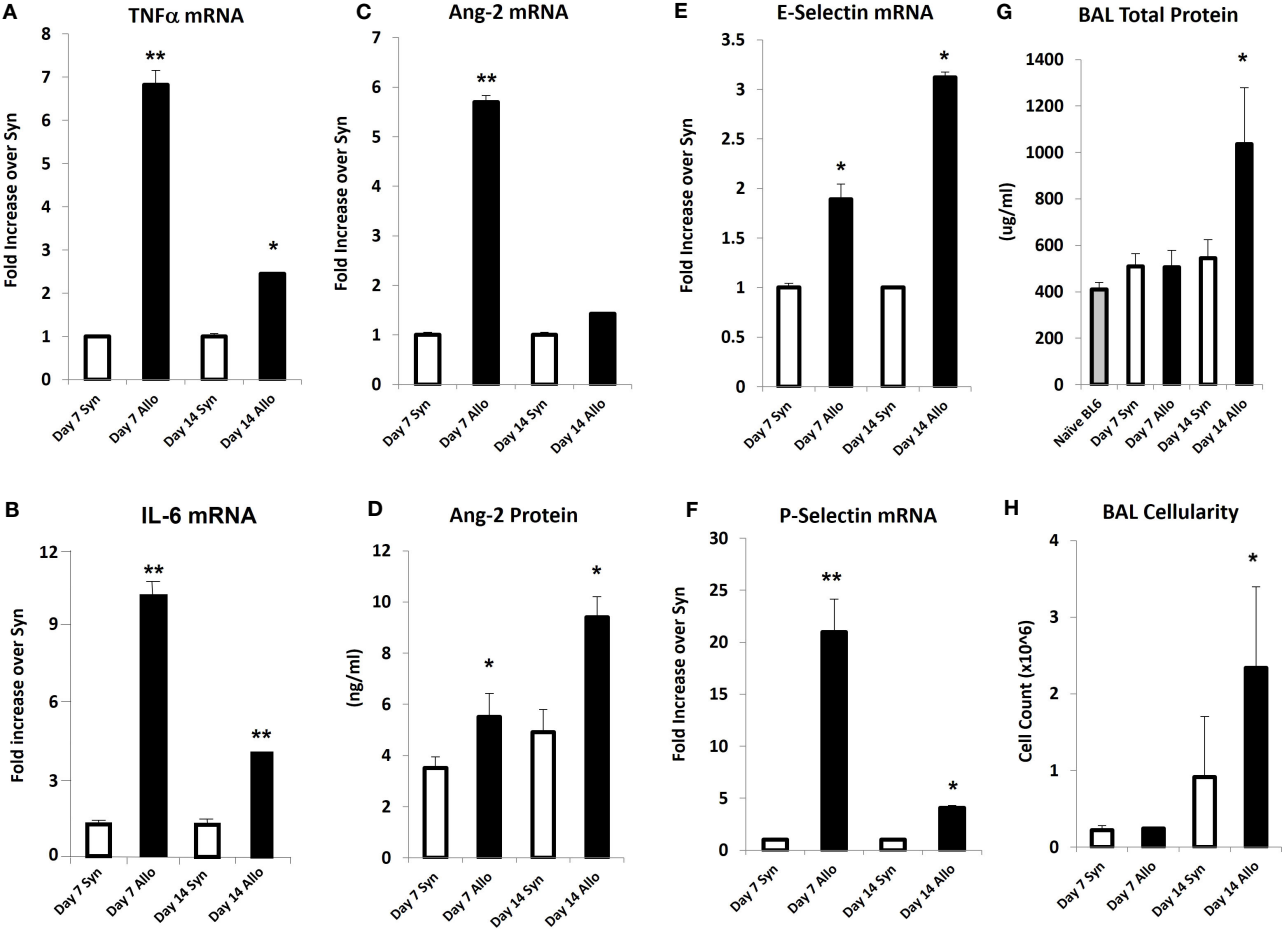
Naïve B6D2F1/J mice underwent syngeneic or allo-BMT as described in *Materials and Methods*. Animals were analyzed at days 7 and 14 after BMT. mRNA analysis of whole lung tissue shows elevations in the expression of TNFα **(A)**, IL-6 **(B)**, Ang-2 **(C)**, E-selectin **(E)**, and P-selectin **(F)** in allo-BMT recipients compared to syngeneic controls. Elevation in Ang-2 mRNA after allo-BMT associates with increased whole lung Ang-2 protein levels **(D)**. BAL was completed as in **Figure 1**. BAL fluid demonstrates elevation in total protein **(G)** and total cellularity **(H)** as early as D14 following allo-BMT (n = 4-5 mice per group per time point; data are from one of two replicate experiments; *p < 0.05; **p < 0.01 compared to Syn at same time point).

Next, mice underwent BMT as described in *Methods*. Mice were harvested on days 7 and 14 after BMT in order to capture patterns of lung inflammation developing prior to histopathologic evidence of lung injury and clinical GVHD. Analysis of whole lung tissue revealed that mRNA expression of inflammatory cytokines (TNFα and IL-6), and Ang-2 along with E- and P-selectin was elevated as early as day 7 in allo-BMT recipients compared to syngeneic controls (**Figures 4A–E**) confirming and extending prior observations showing elevated protein levels of TNFα and IL-6 along with evidence for down-stream signaling in the lungs of mice and humans with IPS (8, 13, 18–20, 34, 36). These changes pre-dated elevations in whole lung Ang-2 and total protein levels along with BAL fluid cellularity (**Figures 4F–H**), indicative of evolving EC activation, injury, and loss of barrier integrity during the development of experimental IPS.

### Defibrotide dose finding assay reveals optimal dose for repeated i.p. injections

**Figure 5:**
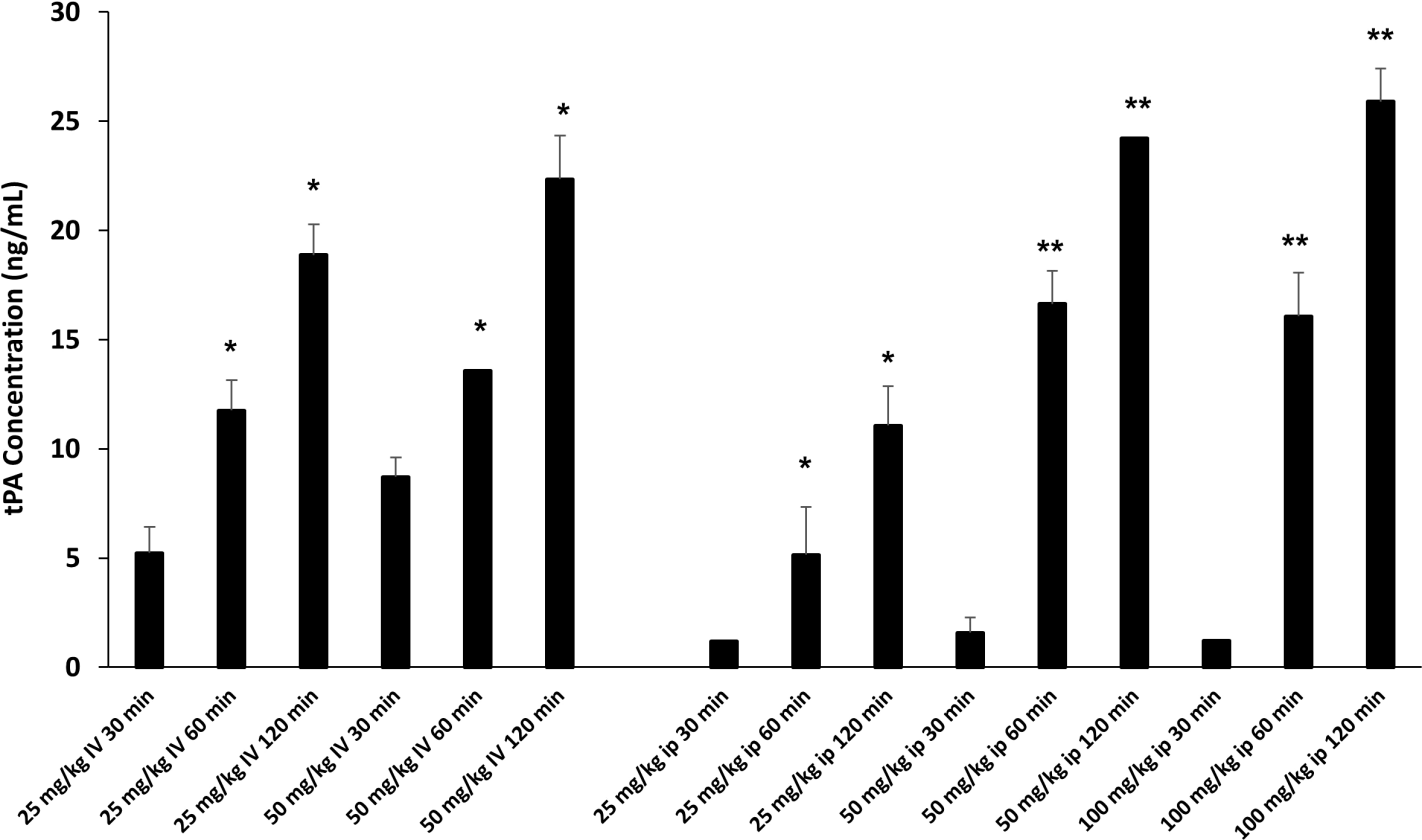
Naïve C57BL/6J mice were injected with DF i.v. or i.p. routes at varying doses (i.v. 25 mg/kg, 50 mg/kg; ip: 25 mg/kg, 50 mg/kg, 100 mg/kg). Mice were terminally exsanguinated via orbital bleed at 30, 60, or 120 minutes thereafter. Plasma was tested for t-PA level by ELISA as described in *Materials and Methods*. A steady rise in t-PA levels was noted at all doses over the 120 minute interval post injection. When DF is given i.p. the peak levels of t-PA at 50 mg/kg and 100 mg/kg are comparable to the levels measured when DF is given at 50 mg/kg i.v. (n = 3 mice per group at each time point; *p < 0.05, **p < 0.01 compared to 30 min time point).

Defibrotide dosing and route of administration varies widely in reported animal studies for various clinical indications (37–40). We were interested in determining the effects of DF on EC activation and injury in each of our acute lung injury models. Intravenous (i.v.) dosing could be considered before and after LPS administration. However, our interest in treating mice during the early inflammatory events (Day 0 to 14) that characterize the development of IPS made repeated i.v. dosing impractical. We therefore sought to compare i.v. versus intraperitoneal (i.p.) dosing of DF to aid in establishing optimal route, dose and schedule. We took advantage of the fact that DF administration leads to an elevation of tissue plasminogen activator (TPA) (41, 42) which in turn inhibits plasminogen activator inhibitor-1 (PAI-1). In this context, a dose finding experiment was conducted using measured TPA protein levels as a marker of DF activity. Naïve C57/BL6 received increasing doses of DF i.v. or i.p. Dose and route included 25 mg/kg and 50 mg/kg i.v., and 25 mg/kg, 50 mg/kg, and 100 mg/kg i.p. Plasma was subsequently collected via terminal exsanguination 30, 60, or 120 minutes later, cryopreserved and tested in batch. As shown in **Figure 5**, increasing TPA levels were measured by ELISA at each time point. When delivered i.v. 50 mg/kg of DF resulted in higher levels than 25 mg/kg at every time point. While 25 mg/kg of DF given i.p. did not result in appreciable levels of TPA compared to i.v. dosing, both the 50 mg/kg and 100 mg/kg i.p. doses ultimately had similar effects to 50 mg/kg delivered i.v. though the TPA elevation was slightly delayed. Therefore, administering DF i.p. was comparable to i.v. dosing and 50 mg/kg i.v. and 50 mg/kg i.p. were chosen as doses for future studies in the ARDS and IPS models respectively (**Figure 5**).

### Defibrotide reduces EC activation and leak induced by LPS administration

**Figure 6:**
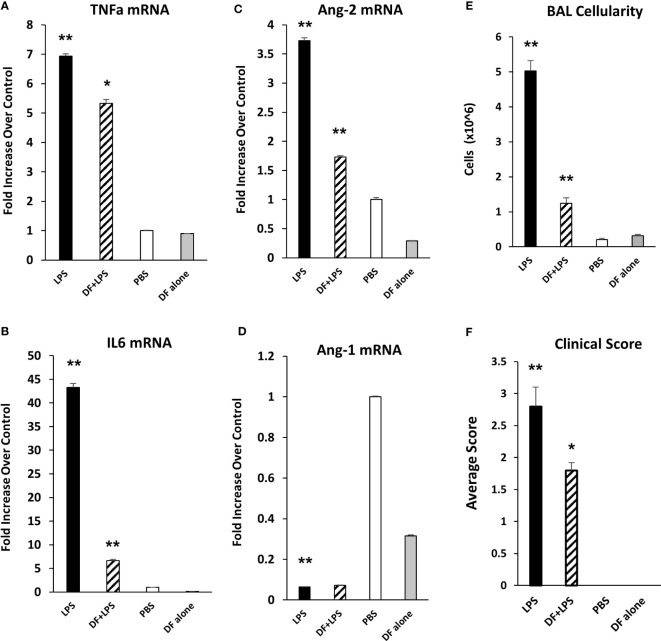
Naïve C57BL/6J mice were injected with LPS as described in **Figure 2**. Mice were randomized to receive either DF 50 mg/kg or PBS i.v. at 1 hour before and 5 hours after LPS administration. Animals were evaluated 12 hours after LPS. Additional controls received PBS or DF only. mRNA analysis of whole lung tissue revealed a reduction in the expression of TNFα **(A)**, IL-6 **(B)** and Ang-2 **(C)** in mice receiving DF + LPS (hashed bars) compared to mice receiving LPS + PBS (solid black bars), whereas Ang-1 expression was modestly restored **(D)**. Administration of DF also reduced BALF cellularity **(E)** and clinical score **(F)** of LPS treated mice (n = 5 mice per group; data are from one of two replicate experiments; *p < 0.05, **p < 0.01 LPS compared to PBS, and DF+LPS compared to LPS).

To determine whether DF could mitigate EC activation and injury incurred during LPS-induced ARDS, mice were injected with DF 50 mg/kg i.v. 1 hour before and 5 hours after exposure to LPS. BAL fluid and lungs were examined at 12 hours after the administration of LPS (DF+LPS). Control groups of mice were exposed to PBS only (PBS), LPS only (LPS), or DF without LPS exposure (DF alone). mRNA analysis of whole lung tissue showed reductions in TNFα, IL-6, and Ang-2 expression in the DF treated group compared to controls receiving LPS alone (**Figures 6A–C**), whereas Ang-1 mRNA and Tie-2 mRNA levels were unaffected by DF administration (**Figure 6D** and data not shown). Modulation of inflammation was associated with marked reduction in total cell count (**Figure 6E**) and overall clinical score (**Figure 6F**) measured in DF treated animals compared to PBS-treated controls.

### Administration of defibrotide early after BMT quiesces EC activation and injury and reduces the severity of IPS

**Figure 7:**
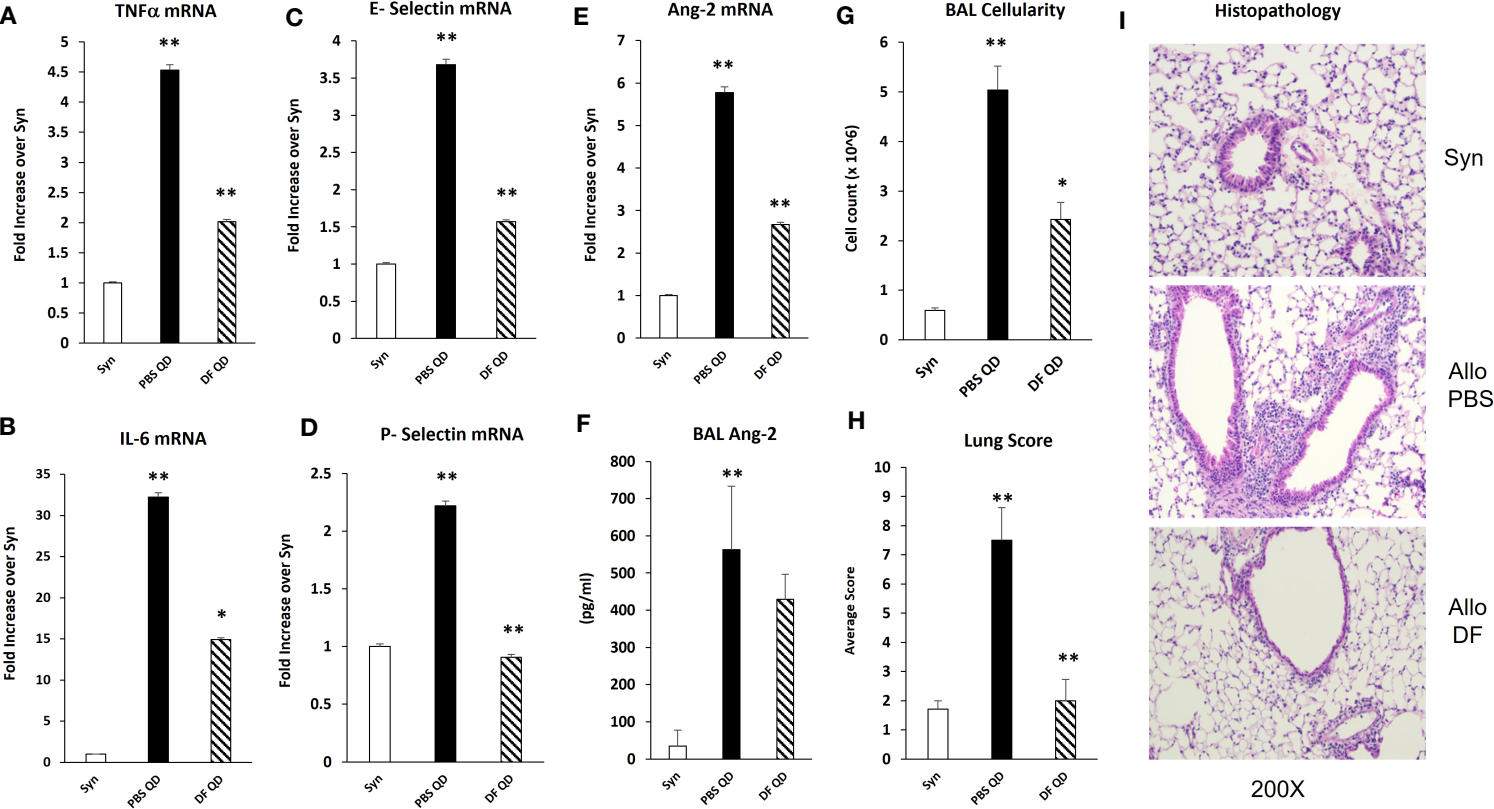
Naïve B6D2F1/J mice underwent syngeneic or allo-BMT as described in **Figure 4**. Mice were treated with DF 100 mg/kg/day or PBS i.p. daily from day -1 through day +14. Mice were harvested on days 18 to 21 after BMT. mRNA analysis of whole lung homogenates shows that the administration of DF to allo-BMT recipients (hashed bar) results in a reduction in the expression of TNFα **(A)**, IL-6 **(B)**, E-selectin **(C)**, P-selectin **(D)**, and Ang 2 **(E)** with a modest decrease in BALF Ang-2 levels **(F)** compared to allogeneic controls receiving PBS (solid black bars). Reductions in the expression of inflammatory cytokines (TNFα and IL-6), selectin molecules and Ang-2 were associated with significant reductions in in BALF cellularity **(G)** and in lung pathology **(H)** compared to allogeneic animals received PBS. Representative photomicrographs of lung histology from each group are shown **(I)**. (n = 4 to 8 mice per group; data are from one of two replicate experiments; *p < 0.05, **p < 0.01 PBS QD compared to Syn, DF QD compared to PBS QD).

**Figure 8:**
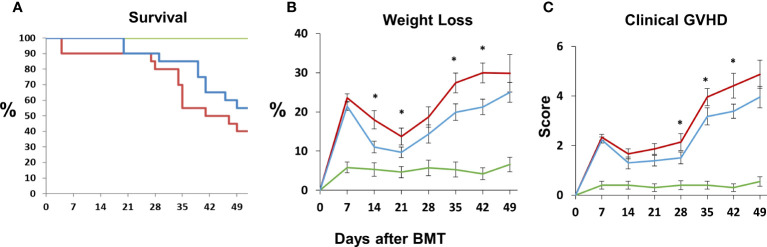
Naïve B6D2F1/J mice underwent syngeneic or allo-BMT as described in **Figure 4**. Allo-BMT recipients were treated with DF 100 mg/kg/day or the equivalent volume of PBS i.p. daily from day -1 through day +14 as in **Figure 7**, and were monitored for signs of systemic GVHD as described in *Materials and Methods*. The administration of DF resulted in modest improvements in survival **(A)**, weight loss **(B)** and GVHD score **(C)** compared to controls. (n = 10 mice (syngeneic) or 20 mice (allogeneic) per group; data are from combined from two replicate experiments; *p < 0.05 DF treated compared to PBS treated allo-BMT recipients. Green line = syngeneic; red line = Allo + PBS; blue line = Allo + DF.

Next, B6D2F1/J mice received BMT from allogeneic (B6) or syngeneic (F1) donors as described in *Methods*. In the first set of experiments, mice were treated with DF 50 mg/kg twice daily i.p., starting the day prior to BMT (Day -1), through 14 days after BMT. A control group was treated with PBS at the same dosing schedule. There was an unacceptably high rate of early death in both groups presumably from repeated trauma to the peritoneal cavity in vulnerable mice early after BMT. Therefore, in subsequent experiments, mice were treated with 100 mg/kg of DF i.p. once per day or a similar volume of PBS i.p. from day -1 to day 14. Mice were sacrificed on days 18 to 21, and lung markers of inflammation were again examined. Analysis of whole lung tissue showed reduction in the mRNA expression of TNFα, IL-6, Ang-2, E-selection, and P-selectin in DF treated mice compared to PBS-treated allogeneic controls (**Figures 7A–F**). Ang-2 protein levels were decreased in the BAL fluid but did not reach statistical significance (**Figure 7F**). Decreased lung inflammation and EC activation directly correlated with reductions in BALF cellularity and pathology score reflective of lung histopathology compared to allo-BMT controls (**Figures 7G–I**). In longer-term BMT experiments, DF was administered from day -1 through day +14 and animals were followed for survival and clinical GVHD as previously defined (34). Allogeneic mice treated with DF in this model had modest improvements in mortality, weight loss, and clinical GVHD score compared to PBS-treated allogeneic mice (**Figure 8**). Notably, despite known anti-thrombotic and fibrinolytic effects of DF, we did not see any systemic or pulmonary bleeding in animals treated with the agent.

### Direct cytokine inhibition and the administration of defibrotide reduces pulmonary EC apoptosis early after allogeneic BMT

**Figure 9:**
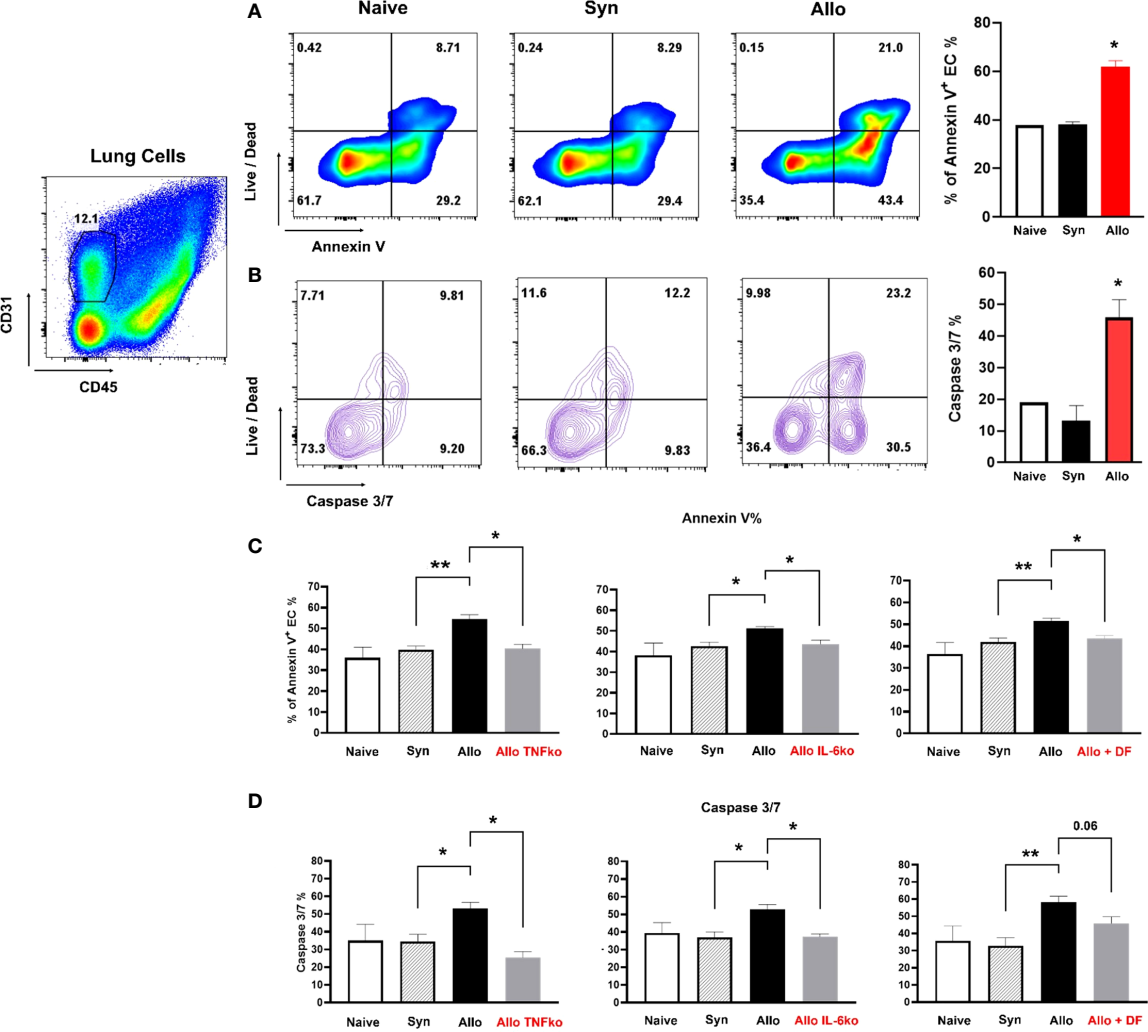
Irradiated B6D2F1 mice underwent syn- or allo-BMT as described in **Figure 4**. Animals were sacrificed at week 1 after BMT and lungs were procured and digested to create a single-cell suspension as described in methods. Cells were then stained with CD31 and CD45. Pulmonary ECs were distinguished from immune cells as being CD31+/CD45-. The presence of EC apoptosis was determined by staining for either Annexin V or caspase 3/7 together with cell viability dyes as described in methods. The extent of pulmonary EC apoptosis observed in allo-BMT recipients was significantly higher compared to both syngeneic and naïve controls using both annexin V **(A)** and caspase 3/7 **(B)**.The absence of TNFα and IL-6 in donor cells each contributed to a reduction in EC apoptosis as measured by Annexin V **(C)** or caspase 3/7 **(D)** in this context. Treatment with DF also reduced EC apoptosis one week after BMT **(C, D)**. Data shown are from 2 replicate experiments n = 6 to 8 mice per BMT group and a total of 4 naïve animals. *p < 0.05, **p < 0.01.

In a final set of experiments, we established an assay to examine evidence for EC apoptosis after BMT using flow cytometric measurements (described in *Methods*). Irradiated B6D2F1 mice again underwent syn- or allo-BMT as described above. Animals were sacrificed at week 1 after BMT and lungs were procured and digested to create a single-cell suspension using a GentleMACS (Miltenyi Biotec) tissue dissociator and a digestion mixture containing collagenase IV (0.05%) and DNase (1mg/ml) as described in *Methods*. Pulmonary ECs (CD31+) were distinguished from immune cells (CD45+) as being CD31+/CD45-. The presence of EC apoptosis was determined by staining for either Annexin V or caspase 3/7 together with cell viability dyes. We found that the extent of pulmonary EC apoptosis observed in allo-BMT recipients was significantly higher compared to both syngeneic and naïve controls using both annexin V (**Figure 9A**) and caspase 3/7 (**Figure 9B**). Given the aforementioned roles of TNFα and IL-6 to clinical and experimental IPS, we next determined the contribution of TNFα and IL-6 producing donor cells to EC apoptosis by using the corresponding gene ko mice as BMT donors. As shown in **Figure 9**, the absence of TNFα and IL-6 in donor cells each contributed to a reduction in EC apoptosis as measured by Annexin V (9C) or caspase 3/7 (9D) in this context. In a final set of experiments, allo-BMT mice were treated i.p. with DF or PBS as before from Day-1 to D+7. DF treatment also reduced EC apoptosis one week after BMT (**Figures 9C, D**).

## Discussion

ARDS and IPS following allo-BMT are significant contributors to MODS, which is a leading cause of morbidity and mortality in pediatric patients (3, 4). Specifically, pulmonary dysfunction remains a significant problem after allo-BMT, limiting successful outcomes (5, 43). Data presented herein demonstrate that established models of IPS and LPS-induced ARDS are associated with EC activation and injury, leading to pulmonary vascular leak and the influx of donor immune cells into the lung (**Figures 2**, **4**). These data extend previous findings and underscore the contribution of TNFα in each setting; TNFα directly contributes to vascular EC injury (8), and regulates the local (pulmonary) chemokine milieu and subsequent influx of donor cells into the lung (13). Moreover, lung damage and dysfunction in mice can be mitigated by neutralizing TNFα (8, 11). Pre-clinical observations led to the development of clinical trials wherein neutralization of TNFα provided therapeutic benefit in pediatric BMT recipients with IPS (18, 44, 45). While the results of these trials were encouraging, not all subjects with lung injury respond to anti-TNFα therapy. The reasons for this remain to be fully elucidated. It is important to note however that IL-6, a cytokine we and others have shown to contribute to IPS in unique preclinical systems, was also elevated in humans with IPS and specifically those patients with a poor response to TNF neutralization (20).

Toward further improving our understanding of disease, plasma samples obtained from patients with IPS treated on prior clinical studies were evaluated by label-free, peptide-based, protein expression analysis. Results revealed striking similarities in inflammation associated with IPS in humans and mice, emphasized the role for the acute phase response (IL-6 and TNFα) signaling pathway, and identified protein candidates that 1) associate with endovascular injury and 2) may predict at time of allo-BMT which subjects will develop lung injury responsive to TNFα-inhibition therapy (36). Additional evaluation of the plasma proteome using antibody microarrays (24) and ELISA revealed mechanistic clues regarding alternative pathways injury and inflammation; elevations in the expression of TNFα and several molecules associated with EC injury and activation, including Ang-2, VCAM-1, and E-selectin, were noted in patients with IPS when compared to unaffected allo-BMT recipients as controls (44) and data not shown. Importantly, VCAM-1 and E-selectin contribute to distinct stages of leukocyte migration into target tissues during inflammation (46, 47).

Angiopoietin-1 (Ang-1) and angiopoietin-2 (Ang-2) are functional antagonists that competitively bind to the EC receptor Tie-2 to regulate vascular integrity: Ang-1 promotes vessel stability, whereas Ang-2 contributes to increased vascular permeability (5, 48–50). Recent studies show that Ang-2 works in part by sensitizing ECs to the effects of TNFα, specifically with respect to TNFα-mediated adhesion molecule expression (51). In data presented herein, evidence for inflammation along with pulmonary EC activation and enhanced expression of Ang-2 is apparent early after LPS administration (**Figure 2**) and regulated in part by TNFα (**Figure 3**). Similar findings are also present after allo-BMT, when they associate with increased expression of P- and E-selectin and precede evidence for lung histopathology and increased levels of total protein (as a measure of EC leak) and cellularity in the broncho-alveolar space (**Figure 4**).

Collectively, these findings support the hypothesis that an endothelial stabilizing agent, such as DF, would lessen EC injury and protect the lung in two acute lung injury models. Defibrotide is a sodium salt mixture of single-stranded oligodeoxyribonucleotides derived from porcine intestinal mucosal DNA. Clinical studies have shown that the administration of DF improves survival in BMT patients with VOD/SOS and MODS (52–56). As alluded to above, VOD/SOS is a serious complication after BMT believed to be driven in large part by damage to sinusoid endothelium (57). Clinically, DF is generally well-tolerated with acceptable rates of adverse events and toxicities (58). Preclinical data suggests that DF stabilizes ECs by inhibiting inflammatory and pro-thrombotic activation, and thereby decreasing endothelial cell activation (27, 28, 57, 59, 60). Pertinent to our studies, DF has been shown to dampen inflammatory activity of monocytes and neutrophils both known contributors to IPS and ARDS. Specifically, Shi and colleagues recently established that DF mitigates pyroptosis-mediated cell death by binding neutrophil extracellular trap (NET)-derived histones in an *in vitro* model of neutrophil-mediated activation and injury of the endothelium (26). In addition, *in vitro* models demonstrate that DF can reduce adhesion molecule expression on ECs or leukocyte adhesion in the context of inflammation engendered by sera from patients with acute GVHD or TNFα respectively (29, 61). Finally, DF protects ECs from LPS- (60) and TNFα-mediated (62) EC cytotoxicity along with chemotherapy- and allogeneic cytotoxic lymphocyte-induced EC apoptosis (63) all of which are likely contributors to clinical and experimental IPS (8, 11, 13, 30, 34, 64). We demonstrate that DF modulates pulmonary EC injury in models of ARDS and IPS; markers of inflammation, including TNFα and IL-6, along with the expression of P- and E-selectin were decreased in mice receiving LPS and in allo-BMT recipients following DF administration (**Figures 6**, **7**). Additionally, animals receiving DF in both models had decreases in Ang-2 levels. These findings suggest that EC stabilization with DF could blunt the pathways that associate with loss of EC barrier function and enhanced leukocyte extravasation into the lungs characteristic of ARDS and IPS (**Figures 6**, **7**). In keeping with this, DF administration also reduced the degree of pulmonary EC apoptosis observed as early as week one post-transplant down to the level of syngeneic and naïve controls (**Figure 9**).

Taken together, EC activation and injury contribute to lung inflammation observed in models of ARDS and IPS. Administration of DF quiesces EC inflammation and reduces leukocyte infiltration and histopathology seen in each model. Consistent with prior work, TNFα and IL-6 are associated with EC injury in each context and specifically contribute to pulmonary EC apoptosis seen by day 7 after BMT. These findings suggest that the addition of DF to cytokine inhibition may work synergistically to reduce EC damage and capillary leak that contributes to pulmonary inflammation and dysfunction, and thereby further improve outcomes in patients with IPS. A role for DF in the context of ARDS secondary to infectious etiologies requires further exploration. To this end, an early phase clinical trial has recently shown that DF administration for Covid-19 ARDS is safe and potentially efficacious (65). Larger studies for this indication are ongoing.

## Data availability statement

The original contributions presented in the study are included in the article/supplementary material. Further inquiries can be directed to the corresponding author.

## Ethics statement

The animal study was reviewed and approved by Johns Hopkins University Animal Care and Use Committee.

## Author contributions

Conception and design (OK, KC), generation, collection and/or assembly of data (OK, YK, EP, H-HF, AH, CL), data analysis and interpretation (OK, YK, EP, H-HF, CL, KC), manuscript writing (OK, YK, KC), financial support (OK, KC). All authors contributed to the article and approved the submitted version.
